# Supplementary material for: Sexual behaviour, changes in sexual behaviour and associated factors among women at high risk of HIV participating in feasibility studies for prevention trials in Tanzania
Source: PLoS One. 2020 Apr 16;15(4):e0231766. doi: 10.1371/journal.pone.0231766 (PMC7162511; doi:10.1371/journal.pone.0231766)
Supplement: Attachment — Submitted filename: PLOS One review.docx [file pone.0231766.s001.docx]

General comments:

- I think it is a fairly useful manuscript that strengthen the existing literature on the importance of HIV prevention trials.

Specific comments
=============
Abstract:

- Methods: Please state the year when these women were enrolled into the feasibility studies and their age category i.e. 18-40 years.
- Conclusions: An important point that biological prevention interventions such as PrEP must be emphasized to complement risk reduction counselling so as to minimize HIV acquisition risk but I think PrEP should be directed more to individuals at highest risk – this should come out clear.

Introduction:

- I can see that the hypothesis of this manuscript is that sexual risk behaviours become less risky over time due to women’s participation in the in prevention trials but it doesn’t come out clear in the last paragraph of the introduction.

Methods:

- In line 119 I would say Chi-squared tests instead of Chi-square.
- You mention that factors that were known to be important predictors for HIV acquisition were then entered into the multivariable model and retained if associated at p<0.05. Can you state a handful of these factors?
- In Poisson regression, please add detail on how you define follow-up time overall and particularly among those who seroconverted. For individuals who seroconverted, did you put seroconversion in the middle of the time interval from last HIV-negative test result to first HIV-positive test result or you used the “alpha-network” method of imputing the seroconversion date multiple times - randomly in the time interval during which the seroconversion took place?
- In Poisson regression, I don’t quite understand your selection of ‘best’ model. It looks like your selective approach/ stringent criteria is more informed by the literature (prior knowledge) more than the data. The sexual behaviours you ended up selecting (number of partners in the last three months, condom use at last sex and high-risk sex in the last three months) seem to confirm that this selective approach is biased towards the literature and we therefore don’t learn anything new here. Instead of keeping the selection entirely selective, can’t you think of a penalised Poisson regression modelling to compensate for the few cases?
- Please explain what you mean by ‘high-risk sex in the last three months’.
- Please further clarify in the text how developed the risk score, I’m finding it difficult to follow. You may want to review this old article: Sullivan LM, Massaro JM, D'Agostino RB, Sr. Presentation of multivariate data for clinical use: The Framingham Study risk score functions. Statistics in medicine. 2004;23(10):1631-60. Epub 2004/05/04. doi: 10.1002/sim.1742. PubMed PMID: 15122742

Results:

- It has to be clear to the reader what it means if a woman reported that they engaged in high risk sex in the past three months (see my related comment in the methods).
- Please define consistent condom use.

Discussion:

- Please state the selective approach you used to build Poisson models as a limitation
