# Supplementary material for: Sexual behaviour, changes in sexual behaviour and associated factors among women at high risk of HIV participating in feasibility studies for prevention trials in Tanzania
Source: PLoS One. 2020 Apr 16;15(4):e0231766. doi: 10.1371/journal.pone.0231766 (PMC7162511; doi:10.1371/journal.pone.0231766)
Supplement: Attachment — Submitted filename: Responses to Reviewers .docx [file pone.0231766.s002.docx]

**RESPONSES TO GENERAL COMMENTS FROM THE REVIWERS**

**Reviewer #1, Comment 1:** It’s an interesting study that needs minor revision The authors used a random intercept logistic regression model to estimate odds ratios for the change in prevalence of certain risky behaviours. However, the justification for this multilevel modelling is not clearly stated. For example, is it to cater for variance between visits and correlation between individual repeated measurements? It would have been appropriate to specify the random intercept logistic regression model used for clarity.

*Response* We thank the reviewer for this important question. Yes, as the reviewer points out, the justification for using Logistic regression with random effect intercepts was to account for correlations between repeated measurements at the multiple visits. A sentence has been added to the manuscript to clarify this as suggested by the reviewer . (Page 6, lines 132-133).

**Reviewer #1,Comment 2:** The authors said “since few women were categorized as having risky sexual behaviour at 12 months, no attempt was made to build a full multivariable model to avoid problems with data sparsity” but it’s not clear how factors associated with risky behaviour at 12 months were identified (table 4 of results).

*Response* We thank the reviewer for this comment. The Table four (4) of results presents the outcome of the Logistic regression model which examines each covariate with its association with the “having risky behaviour at 12 months” (outcome of interest). Following the univariate association, each covariate was adjusted for the effect of the “baseline behaviour”. While this is not a full multivariable model, each covariate was only adjusted for the effect of the baseline behaviour. Therefore, the presented adjusted odds ratios are “partially” adjusted as they do not take into account the effect of the other behaviours. (Page 7,line 162-166).

We have revised the manuscript in the section quoted by the reviewer so as to improve clarity of the analyses performed. A footnote in Table 4 explains that the estimated odds ratio are adjusted only for the baseline sexual behaviour risk score.

**Reviewer #1, Comment 3:** Lastly, self-reported changes in risky behaviour is such a highly subjective matter prone to bias especially where the respondent is aware of the purpose of the study. This may have overestimated the effect.

*Response* We agree with the reviewer that self-reported behaviours are prone to reporting bias i.e the social desirability bias. It is for this reasons that we have acknowledge and discussed this as a study limitation and how it may have overestimated the reported reduction of risky behaviour.

In the discussion section, we have also highlighted the several attempts made to minimize this bias during study design and data collection. To underscore the effect of this bias on our study findings, we have reviewed and made necessary additions to this section in the manuscript. (Page 20, line 311-317).

**Reviewer #2 comment 1:** The grammar is largely correct except in a few places where minor corrections are needed. The authors need to make some corrections to the few typos.

*Response* We thank the reviewer for pointing this. We have reviewed the entire manuscript and take note of the grammar and typographical errors. We have corrected the errors and proof-read the final version of the manuscript.

**Reviewer #2 comment 2:** Statistical analysis is clear except in Poisson regression.

*Response* We take note of the reviewer’s concern on the clarity of the Poisson regression analysis performed. We have reviewed this section in detail and revised the paragraph to improve clarity. (Page 7, line 138-154).

**RESPONSES TO SPECIFIC COMMENTS FROM REVIEWER 2**

**ABSTRACT:**

**Comment #1**: Methods: Please state the year when these women were enrolled into the feasibility studies and their age category i.e. 18-40 years.

*Response:* Thank you for this suggestion. We have revised the abstract and included this information in the method section of the abstract. (Page 2, line 27).

**Comment #2**: Conclusions: An important point that biological prevention interventions such as PrEP must be emphasized to complement risk reduction counselling so as to minimize HIV acquisition risk but I think PrEP should be directed more to individuals at highest risk – this should come out clear.

*Response:* Thank you for this comment. We concur with need to emphasize that PrEP should target particular individuals at the highest risk of HIV acquisition. We have revised the conclusion and added this. (Page 2-3, line 46-49).

**INTRODUCTION:**

**Comment #3**: I can see that the hypothesis of this manuscript is that sexual risk behaviours become less risky over time due to women’s participation in the prevention trials but it doesn’t come out clear in the last paragraph of the introduction.

*Response:* We thank the reviewer for this suggestion. A statement on the study’s hypothesis has been added in the last paragraph of the Introduction. (Page 4, line 75-76).

**METHODS:**

**Comment #4** :In line 119 I would say Chi-squared tests instead of Chi-square.

*Response:* Thank you for pointing this out. This has been corrected. (Page 6, line 125 and Page 14, line 213-214).

**Comment #5**: You mention that factors that were known to be important predictors for HIV acquisition were then entered into the multivariable model and retained if associated at p<0.05. Can you state a handful of these factors?

*Response:* We thank the reviewer for this comment. When building and selecting the best multivariable Poisson regression model, a decision was made to have a model that includes covariates measuring condom use and number of partners. These were regarded as important predictors of HIV acquisition in the study population.

**Comment #6**: In Poisson regression, please add detail on how you define follow-up time overall and particularly among those who seroconverted. For individuals who seroconverted, did you put seroconversion in the middle of the time interval from last HIV-negative test result to first HIV-positive test result or you used the “alpha-network” method of imputing the seroconversion date multiple times - randomly in the time interval during which the seroconversion took place?

*Response:* Thank you for this comment. Seroconversion was assumed to be midpoint between last HIV negative and HIV-positive test results. Since women were only followed for 12 months and had short intervals between tests i.e tested for HIV every 3 months, any bias on estimating seroconversion date is likely to be minimal^[1]^.

Furthermore, the aim of the analysis was not estimate HIV incidence but rather to construct a score for “risky behaviour”, there was minimal concern of the potential bias using the midpoint method for estimating seroconversion.

We have revised the manuscript and added a description of how follow-up time and seroconversion date were defined. (Page 6 line 139-141).

**Comment #7**: In Poisson regression, I don’t quite understand your selection of ‘best’ model. It looks like your selective approach/ stringent criteria is more informed by the literature (prior knowledge) more than the data. The sexual behaviours you ended up selecting (number of partners in the last three months, condom use at last sex and high-risk sex in the last three months) seem to confirm that this selective approach is biased towards the literature and we therefore don’t learn anything new here. Instead of keeping the selection entirely selective, can’t you think of a penalised Poisson regression modelling to compensate for the few cases?

*Response:* We thank the reviewer for this comment and take note of the concerns in Poisson regression modelling. However, the purpose of this analysis was not to develop a predictive model for HIV incidence or get a fully adjusted estimate of factors associated with HIV incidence- where a penalised regression might have been justified because of the small number of seroconversions.

Instead, we intended to construct a score for risky sexual behaviour that could then be used to investigate whether women’s behaviour changes over time. To create this score, we assigned points to covariates that we know to be associated with sexual risk.

**Comment #8**: Please explain what you mean by ‘high-risk sex in the last three months’.

*Response:* We thank the reviewer for this comment. “High-risk sex” was a composite variable generated by combining women reporting a history of rape, sex during menses or anal sex in the past three months. This explanation of how this composite variable was constructed is included in line 124-125, page 6. We have also included a description of this variable in each table in the table footnotes.

**Comment #9**: Please further clarify in the text how developed the risk score, I’m finding it difficult to follow. You may want to review this old article: Sullivan LM, Massaro JM, D'Agostino RB, Sr. Presentation of multivariate data for clinical use: The Framingham Study risk score functions. Statistics in medicine. 2004;23(10):1631-60. Epub 2004/05/04. doi: 10.1002/sim.1742. PubMed PMID: 15122742.

*Response:* We thank the reviewer for this comment and the suggested reference. Of note, this analysis was not aimed at developing a predictive model that could be used to identify women at high risk of HIV. Even so, our analysis used a similar logic as in the suggested reference to assign ‘points’ to each covariate that we know to be associated with sexual risk.

**RESULTS:**

**Comment #10:** It has to be clear to the reader what it means if a woman reported that they engaged in high risk sex in the past three months (see my related comment in the methods).

Response: We thank reviewer for pointing this out. A statement on how this

composite variable was generated is found in the statistical analysis section, line 124-125, page 6.

We have also included an explanation on how the variable was generated in the footnote of every table where it appears.

**Comment #11**: Please define consistent condom use.

Response: Thank you for this comment. Frequency of condom use with regular partners and/or non-regular partners was categorized as “consistent” denoting women who reported always using condom, “inconsistent” for women reporting to sometimes or often use condoms and “never” for those reporting not using condoms.

This definition of the categories of condom use frequency has been added in the statistical analysis section (Page 6, line 121-123).

To ensure uniformity, the term “always” which was used to denote “consistent” condom use has been omitted and replaced with the term “consistently”.

**Discussion:**

**Comment #12:** Please state the selective approach you used to build Poisson models as a limitation.

Response: We thank the reviewer for this suggestion. We have added point to discussion of the study limitations (Page 21, line 335-339).

************************

1. Vandormael A, Dobra A, Barnighausen T, de Oliveira T, Tanser F. Incidence rate estimation, periodic testing and the limitations of the mid-point imputation approach. (1464-3685 (Electronic)).
